# The Rise, Fall, and Rethink of (Fluoro)quinolones: A Quick Rundown

**DOI:** 10.3390/pathogens14060525

**Published:** 2025-05-24

**Authors:** Célia Fortuna Rodrigues, Francisco Silva

**Affiliations:** 1 Associate Laboratory i4HB—Institute for Health and Bioeconomy, University Institute of Health Sciences—CESPU (IUCS-CESPU), 4585-116 Gandra PRD, Portugal; 2 UCIBIO—Applied Molecular Biosciences Unit, Translational Toxicology Research Laboratory, University Institute of Health Sciences (1H-TOXRUN, IUCS-CESPU), 4585-116 Gandra PRD, Portugal; 3 LEPABE—Laboratory for Process Engineering, Environment, Biotechnology and Energy, ALiCE-Associate Laboratory in Chemical Engineering, Faculty of Engineering, University of Porto, Rua Dr. Roberto Frias, 4200-465 Porto, Portugal; 4 Department of Pharmaceutical Sciences, University Institute of Health Sciences—CESPU (IUCS-CESPU), 4585-116 Gandra PRD, Portugal

**Keywords:** quinolones, resistance to antimicrobials, (fluoro)quinolones, pharmacokinetics, spectrum of activity

## Abstract

Antibiotics have revolutionized medicine, with (fluoro)quinolones emerging as one of the most impactful classes of antibacterial agents. Since their introduction, four generations of (fluoro)quinolones have been developed, demonstrating a broad spectrum of activity, favourable pharmacokinetics, and clinical efficacy. However, the rise of multidrug-resistant pathogens has posed significant challenges to their continued effectiveness, particularly in healthcare settings. Among the main resistant species, *Staphylococcus aureus*, particularly methicillin-resistant strains (MRSA), *Klebsiella pneumoniae*, *Enterococcus* spp. (*E. faecium* and *E. faecalis*), *Campylobacter* spp., and *Acinetobacter baumannii* are the most important. This critical literature review provides an updated perspective on (fluoro)quinolones (old and new), encompassing their spectrum of activity, pharmacokinetics, mechanisms of resistance, and the role of antimicrobial stewardship in preserving their utility, to address the growing threat of resistance.

## 1. Introduction

The development of synthetic antibiotics of the quinolone group started in the 1960s. Quinoline and quinolone antibiotics have gained significant attention for their tremendous potential in fighting epidemics [1,2]. These synthetically modified compounds, derived from quinine, feature two-ring structures with various substitutions and are FDA-approved for treating numerous ailments including urinary tract infections, malaria, and respiratory infections [1,2]. They work by inhibiting bacterial nucleic acid synthesis through interruption of DNA gyrase and topoisomerase IV enzymes, and have shown promise against viral diseases like SARS, Zika, Ebola, dengue, and COVID-19 by inhibiting viral entry and replication while modulating immune responses [1,2].

Up to the 2020s, four generations of well-known and highly efficient (fluoro)quinolones have been developed and successfully introduced into clinical practice [3]. Those synthetic antimicrobial agents have revolutionized the treatment of a wide range of bacterial infections due to their broad spectrum of activity, high potency, and favourable pharmacokinetic properties [4]. These drugs work by inhibiting the two bacterial enzymes DNA gyrase (topoisomerase II) and topoisomerase IV, which are essential for bacterial DNA replication and transcription. The clinical applications of (fluoro)quinolones span various infections, including respiratory, urinary, gastrointestinal, and even more serious conditions such as hospital-acquired pneumonia and sepsis [5,6]. Conversely, the widespread and sometimes indiscriminate use of (fluoro)quinolones has led to the emergence of drug-resistant bacterial strains, posing a significant challenge to the healthcare system [4,7]. One of the main issues is presently related to the ESKAPE pathogens—*Enterococcus faecium*, *Staphylococcus aureus*, *Klebsiella pneumoniae*, *Acinetobacter baumannii*, *Pseudomonas aeruginosa*, and *Enterobacter* spp. They represent the most significant threats in antimicrobial resistance globally, causing most hospital-acquired infections with high morbidity and mortality rates [8]. These bacteria are characterized by their remarkable ability to “escape” the effects of antibiotics through multiple resistance mechanisms, including biofilm formation, enzyme production, efflux pump overexpression, and target site modifications [9]. Summarizing, *Enterococcus faecium* exhibits intrinsic resistance to multiple antibiotics, with vancomycin-resistant enterococci (VRE) posing a particularly serious clinical challenge in healthcare settings [10]; methicillin-resistant *Staphylococcus aureus* (MRSA) continues to be a major public health concern, with community-acquired MRSA strains now widely prevalent alongside hospital-acquired variants [11]; *Klebsiella pneumoniae* has emerged as a critical threat due to the spread of carbapenemase-producing strains (KPC), which severely limit treatment options for infected patients [12]; *Acinetobacter baumannii* has become notorious for its environmental persistence and rapid acquisition of resistance, with extensively drug-resistant (XDR) and pandrug-resistant (PDR) strains increasingly reported worldwide [13]; *Pseudomonas aeruginosa* demonstrates intrinsic resistance to multiple antibiotics through its remarkably impermeable outer membrane and constitutive expression of efflux pumps, making infections particularly challenging to treat [14]; and *Enterobacter* spp., particularly *Enterobacter cloacae*, have gained increasing attention due to their ability to overexpress AmpC β-lactamases and develop resistance during antimicrobial therapy [15]. The World Health Organization has classified many ESKAPE pathogens as critical or high priority for research and development of new antibiotics, emphasizing the urgent need for novel therapeutic approaches to combat these formidable bacterial adversaries [16].

Resistance to (fluoro)quinolones can arise through various mechanisms, including mutations in the target enzymes, reduced permeability, efflux pump overexpression, and target protection mechanisms [4,17]. The transfer of resistance determinants via mobile genetic elements further exacerbates the problem, allowing for rapid dissemination of resistance across different bacterial species. Factors such as foreign travel, local usage of (fluoro)quinolones in animal husbandry, and the testing practices of microbiology laboratories contribute to the prevalence of resistance [4,17].

Reported treatment failures underscore the importance of antimicrobial stewardship programmes in managing these infections [18,19,20,21,22,23,24,25,26,27] to optimize the use of antimicrobials in general, prevent the emergence and spread of resistant pathogens, and ensure the continued effectiveness of these crucial therapeutic agents. Strategies may include the development of evidence-based prescribing guidelines, the promotion of appropriate use of (fluoro)quinolones, the implementation of surveillance systems to monitor resistance trends, the training of healthcare providers and the general public’s education on the importance of responsible antibiotic use [4,17,28,29]. In this review, among other points, we try to address structure–activity relationships (SAR), targets, mechanisms of action/resistance/tolerance of (fluoro)quinolones, and where we are presently in antimicrobial stewardship of these drugs.

## 2. (Fluoro)quinolones: Main Molecules, Structures, and Applications

### 2.1. Medicinal Chemistry

Quinolones have been studied for more than half a century. Despite their long history, quinolones remain a focus in medicinal chemistry due to the extensive opportunities for chemical modifications, which can lead to significant improvements in the pharmacokinetic and pharmacodynamic properties of the original compounds, opening broad horizons for both chemical and biological investigations [30,31,32,33,34].

**Figure 1:**
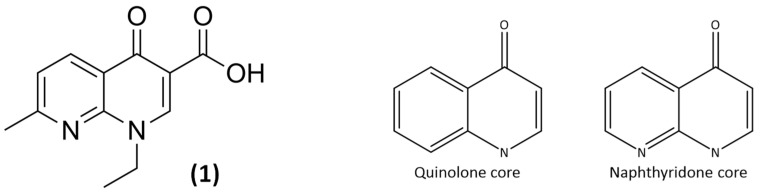
Nalidixic acid, the very first quinolone (**1**), and quinolone versus naphthyridone core.

The clinical and scientific interest in these molecules started with the discovery in the early 1960s of nalidixic acid (Figure 1) (1-ethyl-7-methyl-4-oxo-1,8-naphthyridin-3-carboxylic acid) by Lesher et al. during research on antimalaria agents [35,36]. Indeed, nalidixic acid discovery marked a breakthrough in medicinal chemistry that led to (fluoro)quinolones, a class of important antimicrobial agents. While (fluoro)quinolones are well-known antibacterials, quinolone-based compounds have broader applications, showing activity against malaria, tuberculosis, fungal, and helminth infections. This versatility makes the quinolone scaffold an area of significant research interest across multiple scientific disciplines [37].

Since then, over 10,000 analogues and derivatives of quinolones have been developed, and these drugs have been used to treat more than 800 million patients, which clearly emphasizes their clinical and historical importance, making them one of the most widely prescribed classes of antibiotics [38]. Following Lesher et al.’s initial publication, PubMed has indexed over 23,000 articles and roughly 2600 reviews on the subject. Specifically, regarding quinolones and anaerobes, approximately 300 articles and 70 review papers have been published [39,40,41,42].

Unlike most antimicrobial agents, which are derived from bacteria or yeasts (fungi), quinolones are synthetic compounds [43,44]. From a chemist’s perspective, quinolone molecules are appealing due to their versatile structure. This structural flexibility allows for diverse modifications, enabling the creation of new, more potent, and safer drugs. These modifications not only enhance antibacterial properties but also potentially introduce other valuable biological activities. Their main structure presents a bicyclic system (quinolone core or naphthyridone core) (Figure 1) [45]. Antibacterial drugs based on quinolone and 1,8-naphthyridone structures are commonly grouped under the broad, often interchangeable terms “quinolone” or “fluoroquinolone”. Additionally, the naphthyridone core is sometimes referred to as an 8-azaquinolone [3,46,47].

**Figure 2:**
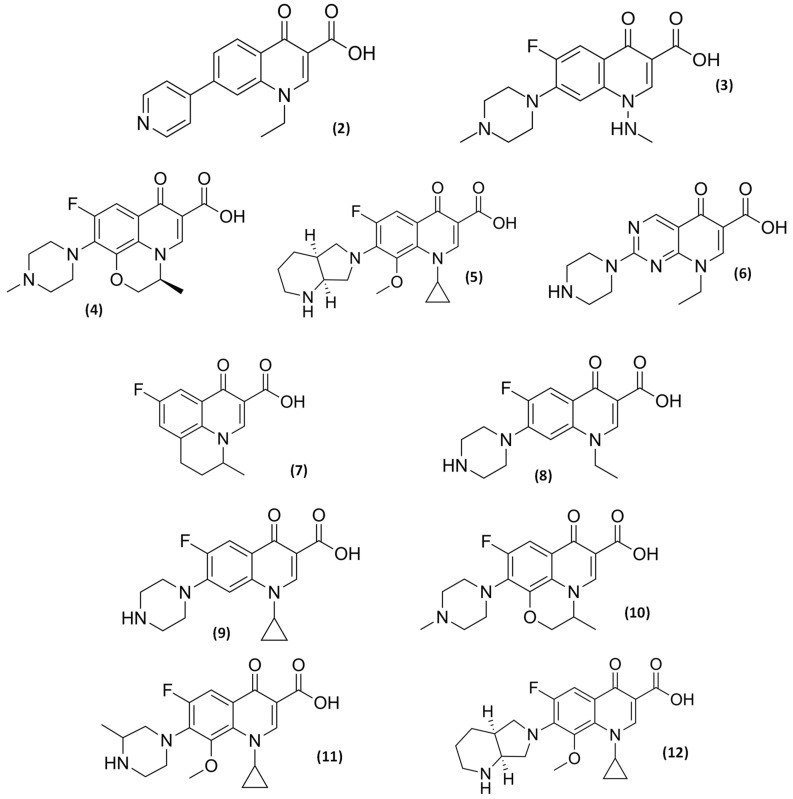
Several quinolones: rosoxacin (**2**), amifloxacin (**3**), levofloxacin (**4**), moxifloxacin (**5**), pipemidic acid (**6**), flumequine (**7**), norfloxacin (**8**), ciprofloxacin (**9**), ofloxacin (**10**), ciprofloxacin (**9**), atifloxacin (**11**), and moxifloxacin (**12**).

The first discovered agent, nalidixic acid (Figure 1), has a naphthyridone core, while other follow-on agents launched, like rosoxacin (**2**) and amifloxacin (**3**), share a quinolone core. Over time, drugs based on both quinolone and naphthyridone cores have been developed and introduced to the market, as there was no clear consensus on whether the naphthyridone core offered significant therapeutic advantages over the quinolone core. The choice of peripheral substituents has proven to be more influential than the core structure in determining antibacterial potency, spectrum of activity, pharmacokinetics, and safety profile. However, the quinolone core does have a technical advantage: the availability of the carbon atom at position 8 (C_8_) for additional substitution, a feature that has been successfully exploited in several successful commercial quinolones such as levofloxacin (**4**) and moxifloxacin (**5**) (Figure 2). While numerous other core variations have been explored and some even marketed (e.g., pipemidic acid (**6**) (Figure 2)), quinolone and naphthyridone-based agents have remained the predominant core structures within this class of antimicrobial agents [45,46,48].

The development of quinolones has been focused on the following aspects: (i) improving pharmacokinetics and pharmacodynamic profile; (ii) enhancing efficacy against antibiotic-resistant bacteria, anaerobes, and atypical pathogens; (iii) minimizing the development of bacterial resistance; (iv) increasing drug selectivity to reduce side effects and improve safety [48]. Modifying the quinolone and naphthyridone cores has been used as the main strategy.

The initial compounds in this class had limited therapeutic applications due to their moderate efficacy against Gram-negative rods. However, significant advancements were made in the late 1970s and 1980s with the synthesis of more potent agents. These improvements were achieved by introducing a fluorine atom at position 6 and a basic amino heterocyclic group (such as pyrrolidine or piperazine) at position 7 of the quinolone or naphthyridone core. These structural modifications substantially increased antimicrobial potency and broadened the spectrum of activity by improving activity against Gram-positive and Gram-negative rods. Flumequine (**7**) was the first patented fluoroquinolone, followed by many others still used today, like norfloxacin (**8**) (Figure 2). Further modification of the quinolone structure resulted in ciprofloxacin (**9**) and ofloxacin (**10**) (Figure 2). Marketed in 1987, ciprofloxacin (**9**) is the most successful and widely used (fluoro)quinolone and based on this molecule, a cyclopropyl group at position 1 was introduced in many newer quinolones, like gatifloxacin (**11**) and moxifloxacin (**12**) (Figure 2) [35,36,39,40,41,42,43,44,47]. Other structural modifications, along with the addition of various substituents at other positions, have resulted in the development of a diverse array of (fluoro)quinolone molecules, each with distinct antimicrobial activity and therapeutic uses.

**Figure 3:**
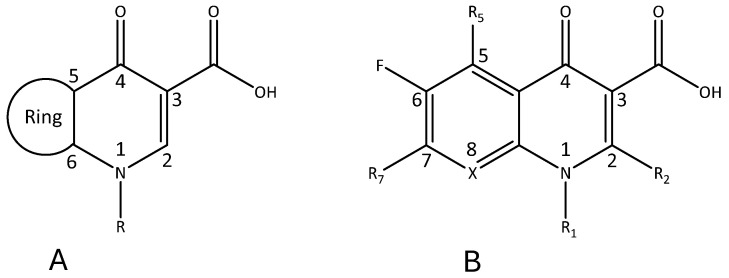
Pharmacophore of quinolones (**A**) and the basic (fluoro)quinolone nucleus for SAR studies (**B**).

SAR studies have provided knowledge about the essential structural features of quinolones. In general, the pharmacophore required for significant antibacterial activity is 4-pyridone-3-carboxylic acid with a ring at 5 and 6 positions (Figure 3) [48].

**Figure 4:**
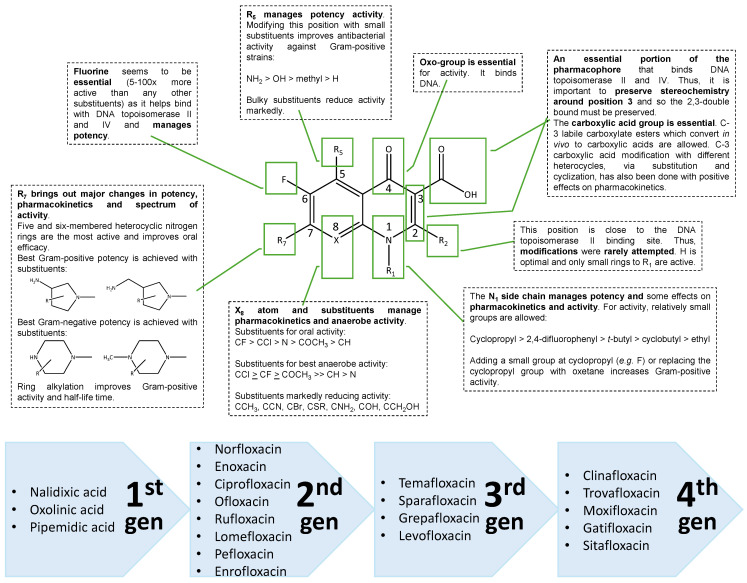
Main conclusions of the SAR studies (adapted from [3,48,51]) and classification of (fluoro)quinolones, with the most representative drugs of each generation (adapted from [35,36,48]).

The oxo group at position 4 and the carboxylic acid group at position 3 are important for binding to target enzymes. The fluorine atom at position 6 gives 5–100 times more active structures than any other group. Substituents R_1_, R_5_, R_7_, and X_8_ control pharmacokinetics, the antimicrobial activity spectrum and potency, thus defining distinct structural features and therapeutic applications (Figure 4) [48,49,50].

Different classification systems for quinolones have been proposed. While the categorization into groups, subgroups, or generations is not universally agreed upon, quinolones are typically divided into four distinct categories (generations) based on their spectrum of antimicrobial activity and pharmacokinetic profile [35,36,48] (Figure 4).

The first generation of quinolones is represented by norfloxacin. The second generation, which includes ciprofloxacin and ofloxacin, exhibits limited or no activity against obligately anaerobic bacteria. Levofloxacin, belonging to the third generation, is the levo-isomer and active component of the chiral molecule ofloxacin. As a result, it shares a similar spectrum of activity with ofloxacin but is twice as potent per unit of mass. In contrast, the fourth-generation quinolones, including sitafloxacin, clinafloxacin, gemifloxacin, garenoxacin, trovafloxacin, moxifloxacin, gatifloxacin, and experimental compounds like WCK 771, and ATB-492, combine improved Gram-positive coverage with maintained Gram-negative activity while also gaining substantial anaerobic coverage. Their expanded spectrum of activity makes them potentially useful for treating a variety of mixed aerobic and anaerobic infections [36,52,53]. Still, due to side effects i.e., photosensivity, hepatic- or cardiac-toxicity, some of the quinolones with good anaerobe activity have been withdrawn from the market in some countries or their development has been terminated prematurely [52,53]. Other quinolones are restricted to the treatment of infections with specific indications [53,54]. Furthermore, recent data show an emergence of quinolone resistance among Bacteroides species. In contrast, a good activity of newer quinolones in anaerobe or mixed infections, i.e., intra-abdominal infections, has also been reported [53,54].

### 2.2. Clinical Applications, Pharmacokinetics, Toxicology, and Resistance Concerns

(Fluoro)quinolones are among the most frequently used antimicrobial agents for the treatment of a wide range of infections in both humans and animals [55]. Their clinical utility is largely attributed to their favourable pharmacokinetic properties, such as excellent gastrointestinal absorption, broad tissue distribution, and good intracellular diffusion [6,7]. However, the extensive and often indiscriminate use of (fluoro)quinolones has led to the emergence of antibiotic-resistant bacterial strains, posing a significant public health concern [6,7].

**Figure 5:**
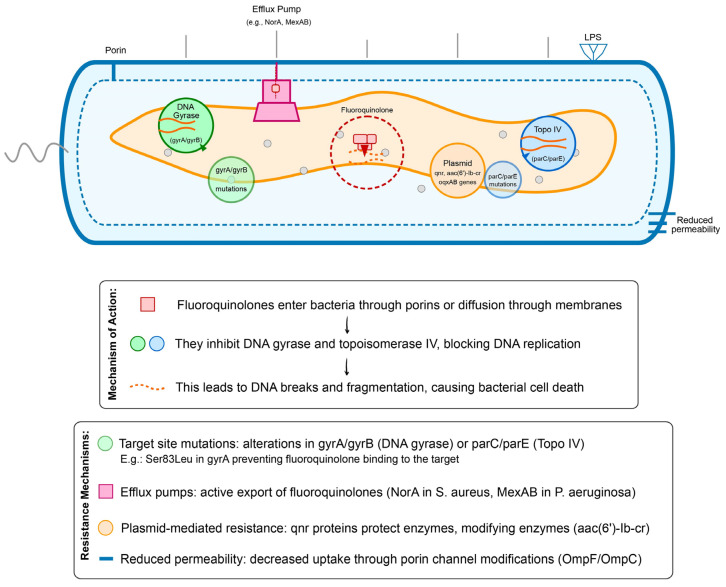
Mechanisms of action and resistance of (fluoro)quinolones.

Research on (fluoro)quinolone antibiotics focuses on two main aspects: direct targeting action causing lesions and indirect toxin accumulation leading to cell death. Drug resistance develops through three mechanisms: mutations at enzyme sites, antibiotic efflux, or plasmid carriage (Figure 5). Despite increasing understanding of quinolones’ relationship with the SOS response involving multiple regulatory genes, questions remain about whether sterilization depends primarily on toxin accumulation, the involvement of toxins beyond ROS generation, how repair protein recF regulates bacterial killing, and how toxin expression is balanced in the body. Given quinolones’ significant bactericidal effects, precautions are needed to reduce the risk of drug abuse [2].

Despite their numerous benefits, the use of (fluoro)quinolones in children has been a subject of ongoing debate due to concerns regarding potential adverse effects [5,7,55]. In general, the pharmacokinetic profile of (fluoro)quinolones is considerably favourable over other antimicrobial compounds. (Fluoro)quinolones are generally well absorbed after oral administration, with peak plasma concentrations typically reached within 1–2 h [7,48]. The oral bioavailability varies within the individual compounds of the class, but is often unaffected by concomitant food intake, making them convenient for patients. (Fluoro)quinolones also exhibit excellent tissue penetration and intracellular accumulation, allowing them to reach high concentrations at the site of infection [7,48]. Their metabolism involves both hepatic and renal pathways, with most of the drug being excreted unchanged in the urine making them suitable to treat urinary tract infections [7,48]. Still, some (fluoro)quinolones, such as ciprofloxacin, may undergo significant first-pass metabolism, leading to lower systemic bioavailability [5]. Therefore, dosage adjustments may be necessary for patients with impaired renal or hepatic function to ensure appropriate drug exposure and minimize the risk of adverse effects.

(Fluoro)quinolones are contraindicated during pregnancy due to observed cartilage damage in juvenile animal studies and potential risks to fetal development [56]. Limited human data suggests potential associations with birth defects, particularly orofacial clefts and cardiac abnormalities in pediatric populations [57]. (Fluoro)quinolones are generally restricted to specific indications where benefits outweigh risks, as they can cause arthropathy and tendinopathy through chondrotoxicity in developing joints [58]. The FDA has strengthened warnings against routine (fluoro)quinolone use in children except for specific scenarios like complicated urinary tract infections and post-exposure prophylaxis for certain infections Recent pharmacovigilance data indicates arthralgia occurs in approximately 3–10% of fluoroquinolone-treated children, with most adverse effects resolving after discontinuation [59].

Although these drugs are characterized by their favourable absorption, distribution, and elimination properties, which contribute to their widespread use in clinical practice, some drug interactions have been reported involving (fluoro)quinolones and other drugs, which may limit their therapeutic efficacy. The oral absorption of (fluoro)quinolones is significantly reduced when co-administered with antacids containing magnesium or aluminum, as well as with other agents like sucralfate. Additionally, the gastric pH can influence the oral absorption of certain (fluoro)quinolones, possibly by affecting their dissolution rate [60].

**Figure 6:**
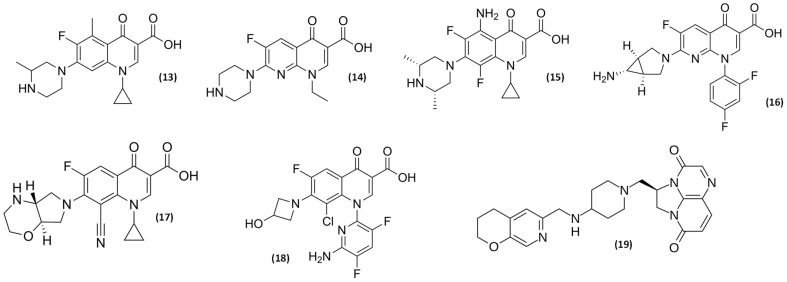
Other quinolones: grepafloxacin (**13**), enoxacin (**14**), sparfloxacin (**15**), trovafloxacin (**16**), finafloxacin (**17**), delafloxacin (**18**), and gepotidacin (**19**).

An important interaction occurs between (fluoro)quinolones and theophylline or other methylxanthines, such as caffeine. This interaction involves isoenzyme CYP1A2 of the cytochrome P-450 pathway and is most pronounced with ciprofloxacin (**9**), norfloxacin (**8**) (Figure 2), and grepafloxacin (**13**) (Figure 6), the use of which can raise serum theophylline concentration to a much greater extent. The clinical consequence of this interaction requires dose reduction and serum concentration monitoring of the xanthines [61,62].

Concurrent administration of some non-steroidal anti-inflammatory drugs (NSAID) and some (fluoro)quinolones (e.g., fenbufen with enoxacin (**14**)) have been associated with the occurrence of important side effects like seizures [48]. Other significant drug interactions mentioned in the literature include elevated serum levels of cyclosporine in case of concomitant use; serum concentration of antineoplastics decrease due to the interaction with ciprofloxacin (**9**) [60]; ciprofloxacin (**9**) and norfloxacin (**8**) serum concentrations increase and decrease in their clearance by interaction with azlocillin, cimetidine, and probenecid [48,60]; sodium bicarbonate, carbonic anhydrase inhibitors and citrates reduce the solubility of norfloxacin (**8**) (Figure 2), and may cause crystalluria [36,48]. Epidemiological studies have linked these drugs to tendinopathy and tendon rupture, peripheral neuropathy, and aortic aneurysm. Safe prescribing requires identifying patients with risk factors for toxicity, promptly discontinuing the drug if adverse reactions occur, and practicing antimicrobial stewardship by only using (fluoro)quinolones when alternatives are unavailable—a practice that also helps limit antibiotic resistance [63].

With few exceptions, the toxicity of (fluoro)quinolones is mild at therapeutic doses and is generally limited to gastrointestinal disturbances such as nausea, vomiting, and diarrhea [48]. The consequences of the adverse effects are not too severe when compared to the benefits of their use.

(Fluoro)quinolones inhibit bacterial DNA synthesis by interfering with DNA gyrase (topoisomerase II) and topoisomerase IV, which are essential enzymes for DNA replication [6]. This mechanism of action contributes not only to their potent bactericidal activity but has also been linked to various adverse effects, including musculoskeletal, neurological, and cardiovascular complications.

**Figure 7:**
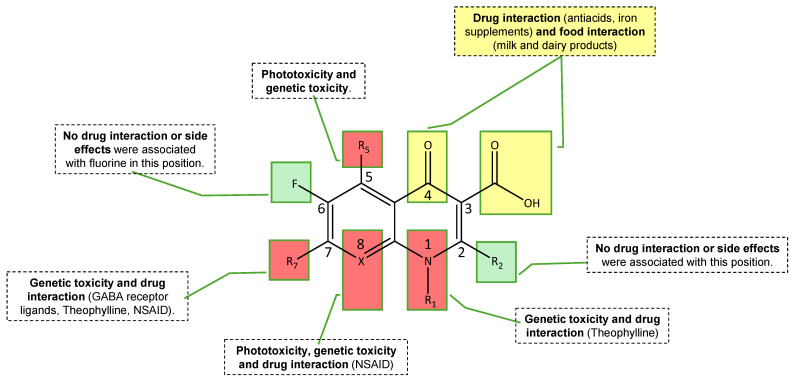
Toxicological effects and drug interactions—concise conclusions from SAR studies [48,61,64].

Some focused adverse events have been described which include skin photosensitivity reactions [48]; CNS effects (e.g., dizziness, insomnia, mood alterations, seizures or hallucination) [48,64], rarely anaphylaxis and agranulocytosis [48,64], and potentially life-threatening dysglycemic reactions [48,62,64]. Also, some concerns have arisen regarding the potential toxicological effects on cartilage and bone development associated with the systemic use of these compounds, particularly in pediatric populations [7,48]. However, recent evidence suggests that prudent use in children may be warranted in certain clinical situations, provided that the potential risks are carefully weighed against the potential therapeutic benefits [7]. Another important serious adverse event reported is prolongation of the QT interval in electrocardiogram (ECG) with some (fluoro)quinolones (sparfloxacin (**15**) and grepafloxacin (**13**) but not with levofloxacin (**4**) and trovafloxacin (**16**)), although this effect is not associated with arrhythmia [48]. Figure 7 shows concise conclusions of toxicological and drug interactions from SAR studies.

One significant safety concern surrounding (fluoro)quinolones use is the development of antibiotic resistance, which can result from the widespread and often inappropriate use of these agents. For instance, (fluoro)quinolones-resistant strains of *Campylobacter* spp. have been documented in up to 50% of clinical isolates, posing a challenge to the effective treatment of *Campylobacter* infections [4,48]. The emergence of resistance has been attributed to factors such as extensive use both in human and veterinary therapy and inadequate antimicrobial susceptibility testing practices.

### 2.3. Older and Novel (Fluoro)quinolones

Nalidixic acid (**1**) (Figure 1), the first synthetic quinolone agent, was discovered in 1962 as a by-product of chloroquine synthesis and was approved for use in the United States in 1964. Since then, the field of (fluoro)quinolones has experienced remarkable advancements, with the development of new generations of these compounds, with enhanced antimicrobial spectrum and improved pharmacokinetic characteristics [5].

Nalidixic acid exhibited antimicrobial activity primarily against Gram-negative bacteria and was mainly used to treat urinary tract infections caused by *Escherichia coli* and *Klebsiella pneumoniae*, among others, with minimal effectiveness against Gram-positive bacteria. Its renal excretion made nalidixic acid well-suited for urinary tract infections. However, its use was restricted to less severe infections due to its low potency, high protein-binding capacity, short half-life, and limited bioavailability [65,66]

The evolution of (fluoro)quinolones has led to the introduction of numerous novel molecules, each with unique structural features and applications. As previously seen, norfloxacin (the first (fluoro)quinolones), introduced in 1986, was followed by ciprofloxacin in 1987, which further expanded the activity of this class of antibiotic agents [5]. The continuous research and development in this field have resulted in the synthesis of even more potent and versatile quinolone compounds, making them indispensable in the management of various bacterial infections. The addition of a fluorine atom to the quinolone ring structure (as seen in the development of norfloxacin in 1980) led to the creation of the (fluoro)quinolone class, which exhibited a broader spectrum of activity [55]. Further modifications, such as the addition of a single carbon atom to norfloxacin, resulted in the synthesis of ciprofloxacin in 1983, which demonstrated increased potency [67,68,69,70]. The latest quinolones exhibit enhanced antibacterial activity against Gram-positive bacteria. As new members are added to the quinolone family, their activity against anaerobic bacteria has also improved. For instance, in the early development of quinolones, ciprofloxacin and ofloxacin were reported to be effective only against *Propionibacterium acnes* and certain strains of *Clostridium perfringens* among Gram-positive bacteria [67,68,69,70].

(Fluoro)quinolones are strongly associated with *Clostridioides difficile* infection (CDI) through multiple mechanisms. These broad-spectrum antibiotics significantly disrupt the protective gut microbiota, creating an ecological niche favourable for *C. difficile* proliferation [71]. Studies demonstrate that (fluoro)quinolone use increases CDI risk by 3–8 fold compared to other antibiotics [72]. (Fluoro)quinolones both trigger new infections and increase recurrence rates in previously infected patients [73]. Risk increases with longer treatment duration and is highest within the first month after (fluoro)quinolone exposure [74]. The molecular mechanism involves fluoroquinolone-induced DNA gyrase mutations in *C. difficile strains,* conferring resistance advantages while preserving virulence factors [75]. Current clinical guidelines recommend avoiding (fluoro)quinolones in patients with previous CDI history or during institutional outbreaks [76].

Recent advancements in (fluoro)quinolone research have focused on structural modifications to combat resistance and reduce toxicity. The development of delafloxacin, an anionic (fluoro)quinolone with enhanced activity in acidic environments, represents a significant breakthrough for treating acute bacterial skin infections and community-acquired pneumonia caused by resistant pathogens [77]. The emergence of newer (fluoro)quinolones like finafloxacin (**17**) (Figure 6) demonstrates improved activity against biofilm-forming bacteria and maintains efficacy under varying pH conditions [78]. On the other hand, computational approaches have led to a better understanding of structure–activity relationships, enabling the rational design of novel (fluoro)quinolone hybrids with activity against ESKAPE pathogens [79]. Pharmacodynamic research has optimized dosing regimens to minimize resistance development while maximizing efficacy, with recent studies confirming that AUC/MIC ratios above 87 significantly improve clinical outcomes [80]. Additionally, innovative delivery systems, including nanoparticle formulations and combination therapy approaches with efflux pump inhibitors have shown promise in restoring (fluoro)quinolone activity against resistant strains [81].

Over the past decade, several new (fluoro)quinolone agents (Figure 6) have been introduced into clinical practice, each offering unique characteristics and targeting specific pathogens. These include delafloxacin (**18**), approved in 2017 for the treatment of acute bacterial skin and skin structure infections, and gepotidacin (**19**), a novel triazaacenaphthylene antibiotic that has shown promise in the treatment of uncomplicated urinary tract infections and gonorrhea [5,7,55].

### 2.4. Tolerance and Resistance Mechanisms Related to (Fluoro)quinolones

**Figure 8:**
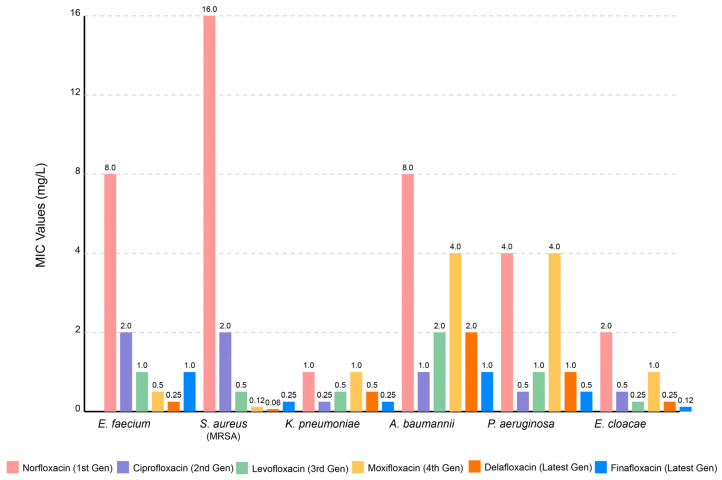
Inhibitory effects of several (fluoro)quinolones on ESKAPE pathogens [90,91,92,93].

One of the key advantages of (fluoro)quinolones is their broad-spectrum activity, which encompasses a wide range of Gram-negative and Gram-positive bacteria, including those that have become resistant to other antibiotic classes [6,7]. This property has contributed to their extensive use in both human and veterinary medicine, leading to concerns about the development of drug-resistant bacteria [4,82]. Resistance to (fluoro)quinolones can arise through multiple mechanisms, including mutations in the target genes encoding DNA gyrase (topoisomerase II) and topoisomerase IV, as well as alterations in membrane permeability and increased efflux pump activity (Figure 8) [4,82].

One of the most well-documented mechanisms of (fluoro)quinolones resistance is the acquisition of mutations in the genes encoding the subunits of DNA gyrase and topoisomerase IV, which are the primary targets of these antibiotics [4,82]. These mutations can lead to reduced binding affinity of the antibiotics to their targets, thereby conferring resistance. Resistance can also occur through the overexpression of efflux pumps, which can actively transport the antibiotics out of the bacterial cell, reducing their intracellular concentration [83]. Additionally, decreased expression of porin proteins in the outer membrane can reduce the influx of (fluoro)quinolones, contributing to resistance [2,52,53].

The emergence of (fluoro)quinolones-resistant bacteria has been observed in various species, including *Campylobacter* spp., with mutations in the gyrA gene, which encodes the A subunit of DNA gyrase [4,28,82,83]. Also, the widespread use of (fluoro)quinolones, particularly in animal husbandry, has been linked to the rise in *Campylobacter* strains exhibiting resistance to these drugs [17]. The emergence of fluoroquinolone-resistant *C. difficile* strains, particularly the hypervirulent NAP1/BI/027 strain, directly correlates with increased (fluoro)quinolone prescribing patterns [84]. Other bacterial species have developed significant resistance to (fluoro)quinolone antibiotics, posing challenges in clinical treatment. *Pseudomonas aeruginosa* demonstrates high-level intrinsic and acquired (fluoro)quinolones resistance through multiple mechanisms, including efflux pumps and chromosomal mutations [85]. *Staphylococcus aureus*, particularly methicillin-resistant strains (MRSA), exhibits increasing (fluoro)quinolone resistance rates, often mediated by target site modifications and plasmid-mediated resistance genes [86], and *Klebsiella pneumoniae* has also emerged as a critical multidrug-resistant pathogen, with carbapenem-resistant isolates frequently showing concurrent (fluoro)quinolone resistance through mutations in gyrA and parC genes [87]. Furthermore, *Enterococcus* spp. (*E. faecium* and *E. faecalis*) displays notable resistance mechanisms, including target site alterations and enhanced efflux pump activity [88]. Finally, *Acinetobacter baumannii* is particularly concerning (one of the WHO critical species [89]), with extensively drug-resistant strains demonstrating high-level (fluoro)quinolone resistance through chromosomal mutations and mobile genetic elements [27]. The latest reports of MIC values of ESKAPE genes are in Figure 8.

In addition to resistance, some bacterial species can also exhibit tolerance to (fluoro)quinolones. Tolerance refers to the ability of bacteria to survive in the presence of an antibiotic without being killed, even though they are not resistant. It is a phenotypic trait where bacteria survive antibiotic exposure without genetic changes, essentially “waiting out” the antibiotic’s effect without growing or dying, typically by entering a metabolically dormant state [94]. This phenomenon can occur due to various mechanisms, such as the formation of persister cells or the activation of stress response pathways [94]. Table 1 summarizes this data.

To address resistance/tolerance, ongoing efforts are focused on the synthesis of new (fluoro)quinolone analogues that can circumvent existing resistance mechanisms and maintain their efficacy against emerging bacterial threats [5,83,87]. The clinical and pharmaceutical analysis of (fluoro)quinolones requires effective analytical procedures for quality control, pharmacodynamic, and pharmacokinetic studies. Various spectrophotometric methods, including visible spectrophotometry, UV spectrophotometry, and derivative spectrophotometry, have been developed and utilized for the determination of these drugs [5].

**Table 1:** Resistance and tolerance mechanisms associated to (fluoro)quinolones.

Feature		Description	Reference(s)
**Resistance**	Main Mechanisms of Resistance	- Mutations in target genes (DNA gyrase and topoisomerase IV) - Reduced binding affinity due to gene mutations - Increased efflux pump activity (actively transports antibiotics out of bacterial cells) - Decreased porin expression (reduces drug influx into bacterial cells)	[4,8,9,19,57,58,64]
	Key Targets for Resistance	- DNA gyrase (gyrA gene mutations) - Topoisomerase IV	
	Species with Most Documented Resistance	- *Campylobacter* spp. (mutations in gyrA gene) - *Klebsiella pneumoniae* (mutations in gyrA and parC genes) - *Enterococcus* spp. (*E. faecium* and *E. faecalis*) (target site alterations and enhanced efflux pump activity) - *Acinetobacter baumannii* (chromosomal mutations and mobile genetic elements)	
	Drivers of Resistance	- Overuse in animal husbandry (linked to the rise of resistant *Campylobacter* strains)	
**Tolerance**	Mechanisms of Tolerance	- Formation of persister cells - Activation of stress response pathways	[18,94]

Meanwhile, several innovative alternative strategies have emerged to optimize (fluoro)quinolone use while addressing resistance concerns. These strategies are summarized in Table 2.

**Table 2:** Alternative strategies to optimize (fluoro)quinolone applications.

Approach	Narrative	Reference(s)
Combination Therapy	Pairing (fluoro)quinolones with complementary antimicrobials has shown synergistic effects against resistant pathogens. The combination of levofloxacin with colistin demonstrates enhanced activity against multidrug-resistant *P. aeruginosa* through increased bacterial membrane permeability.	[95,96,97,98,99]
Efflux Pump Inhibitors	Co-administration of efflux pump inhibitors with (fluoro)quinolones reverses resistance in ESKAPE pathogens. Compounds like phenylalanine-arginine β-naphthylamide (PAβN) restore ciprofloxacin susceptibility in resistant *A. baumannii* by blocking efflux mechanisms.	[99,100,101,102,103]
Nanocarrier Delivery Systems	Lipid and polymer nanoparticles improve (fluoro)quinolone pharmacokinetics and target delivery. Ciprofloxacin-loaded PLGA nanoparticles demonstrate enhanced biofilm penetration and increased antibacterial activity against intracellular pathogens.	[104,105,106]
Biofilm-Targeting Strategies	Modified (fluoro)quinolones with anti-biofilm properties address persistent infections. Ciprofloxacin conjugated with iron chelators disrupts established biofilms in chronic respiratory infections through coordinated antimicrobial and iron sequestration mechanisms.	[107,108,109,110]
(Fluoro)quinolone Hybrids	Novel hybrid molecules combining (fluoro)quinolone pharmacophores with other antimicrobial moieties enhance spectrum and potency. Ciprofloxacin-triazole hybrids demonstrate improved activity against (fluoro)quinolone-resistant *S. aureus* while maintaining Gram-negative coverage.	[111,112,113,114]
Photodynamic Antimicrobial Therapy	Light-activated (fluoro)quinolone derivatives generate reactive oxygen species for enhanced bactericidal activity. Photoactivated ciprofloxacin conjugates demonstrate efficacy against resistant pathogens through mechanisms distinct from conventional antibiotic action.	[114,115,116,117,118]
Pulmonary and Topical Delivery Systems	Alternative administration routes optimize (fluoro)quinolone delivery to specific infection sites. Inhaled levofloxacin formulations achieve higher local concentrations in respiratory infections while minimizing systemic exposure and toxicity.	[119,120,121,122,123]
pH-Responsive Delivery Systems	Smart delivery platforms release (fluoro)quinolones specifically under infection-associated acidic conditions. pH-sensitive hydrogels delivering delafloxacin provide enhanced activity in the acidic microenvironment of abscesses and infected tissues.	[78,124]

### 2.5. Where Are We Presently on (Fluoro)quinolone Stewardship?

**Figure 9:**
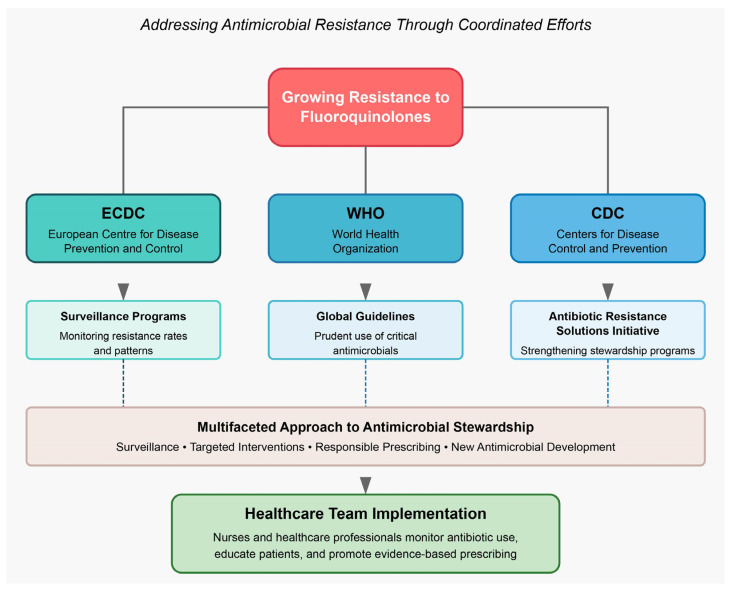
Summary of the present global (fluoro)quinolone stewardship initiatives.

As previously discussed, the widespread use and misuse of (fluoro)quinolones have led to the emergence of drug-resistant strains, raising significant concerns about their long-term efficacy. To address this growing challenge, several global health organizations have developed initiatives and guidelines to promote responsible (fluoro)quinolone stewardship, with a multifaceted approach (Figure 9). This includes continued surveillance of resistance patterns, targeted interventions to reduce inappropriate antibiotic use, and the development of new antimicrobial agents to combat the emergence of resistant strains.

The European Centre for Disease Prevention and Control (ECDC) has been at the forefront of (fluoro)quinolone stewardship efforts [125]. They have emphasized the importance of monitoring resistance rates and implementing targeted interventions to curb the spread of resistant bacteria. Furthermore, the World Health Organization (WHO) has also recognized the need for improved antimicrobial stewardship, issuing guidelines that encourage the prudent use of (fluoro)quinolones and other critical antimicrobials [4]. In the United States, the Centers for Disease Control and Prevention (CDC) has launched the Antibiotic Resistance Solutions Initiative [126], which focuses on strengthening antibiotic stewardship programmes nationwide. The rise of multidrug-resistant pathogens, particularly in the healthcare setting, has heightened the need for effective antibiotic stewardship strategies. Hospital-based antimicrobial stewardship programmes targeting (fluoro)quinolone restriction have shown up to 60% reductions in CDI incidence [Nurses, as integral members of the healthcare team, play a crucial role in implementing antibiotic stewardship practices, such as monitoring antibiotic use, educating patients, and promoting adherence to evidence-based prescribing guidelines [126,127].

## 3. Bibliographic Search

A bibliographic search for this narrative review was conducted using PubMed, Science Direct, and Web of Science databases with keywords and MeSH terms related to “quinolones”, “(fluoro)quinolones”, “bacteria”, and “antimicrobial resistance + (fluoro)quinolones”.

Studies were included if they: focused on (fluoro)quinolones (including pharmacological properties, mechanisms of action, clinical applications, resistance patterns, or adverse effects); were original research, systematic reviews, or meta-analyses; were published within the last decade (2014–2024) unless considered foundational; were written in English; and involved human, animal, or in vitro models relevant to (fluoro)quinolones’ clinical or pharmacological context.

Studies were excluded if they: did not directly address (fluoro)quinolones or their clinical and pharmacological context; were opinion pieces, editorials, conference abstracts, or lacked original data; were not in English (unless reliably translated); were published before 2013 (unless seminal contributions); or contained incomplete data, unclear methodologies, or insufficient detail to evaluate findings.

## 4. Conclusions

(Fluoro)quinolones were primarily discovered in the 60s and are one of the most important classes of antimicrobials, since they possess a broad spectrum of activity against both Gram-negative and Gram-positive bacteria, and recent molecules possess enhanced antimicrobial properties and diverse applications. (fluoro)quinolones have many favourable properties, including excellent bioavailability when given orally, good tissue penetrability and a relatively low incidence of adverse and toxic effects.

On the other hand, the widespread use of (fluoro)quinolones has led to concerns about the development of antimicrobial resistance, which can compromise their clinical efficacy. Therefore, prudent prescription of (fluoro)quinolones, along with the implementation of antimicrobial stewardship programmes, has been and is still crucial to preserve the clinical utility of this important class of antibiotics.

The continuous efforts to develop new and improved (fluoro)quinolones compounds possessing enhanced antimicrobial properties are crucial in addressing the growing challenge of antibiotic resistance and ensuring the effective management of bacterial infections [5,6,7,55].
